# Sponsorship by food and beverage companies in soccer: an analysis of the 2019 Copa América

**DOI:** 10.11606/s1518-8787.2022056003491

**Published:** 2022-05-18

**Authors:** Larissa Cardoso de Miranda Araujo, Juliana de Paula Matos, Paula Martins Horta

**Affiliations:** I Universidade Federal de Minas Gerais Departamento de Nutrição Belo Horizonte MG Brazil Universidade Federal de Minas Gerais. Departamento de Nutrição. Belo Horizonte, MG, Brazil

**Keywords:** Soccer, Food Industry, Financial Support, Food and Beverages, Marketing

## Abstract

**OBJECTIVE:**

To identify the sponsorship by food and beverage companies of the teams participating in the 2019 Copa América Soccer Cup and associate this sponsorship with characteristics of the teams and their respective countries.

**METHODS:**

The sponsors of the 10 teams participating in the 46th edition of the Copa América were identified. These entities were classified into (i) food companies, (ii) alcoholic beverage companies, and (iii) other segments. The food companies were classified according to their products, according to the NOVA classification. In addition, data on the number of titles previously won by the teams in the Copa America and the World Cup were obtained, as well as data on the countries’ Human Development Index, annual *per capita* sales of ultra-processed foods, and annual *per capita* consumption of alcoholic beverages.

**RESULTS:**

A total of 89 sponsorships were identified for the 10 teams studied, some of these supporting two or more teams. Eighteen percent of the sponsors were food companies, with 12.4% being ultra-processed foods. The alcoholic beverage category represented 7.9% of the sponsors. Ultra-processed beverage and alcoholic beverage companies sponsored seven of the 10 teams studied. We noted higher participation of ultra-processed foods company sponsors in teams from countries with higher Human Development Index, sales of ultra-processed foods, and number of Copa América and World Cup titles. The sponsorship by alcoholic beverage companies was higher for teams from countries with lower Human Development Index, alcohol consumption, and number of Copa América and World Cup titles.

**CONCLUSION:**

A significant presence of ultra-processed food and alcoholic beverage companies as sponsors of South American soccer teams was noted, along with the fact that sport performance characteristics of the teams and socioeconomic and market issues of the countries are associated with the occurrence of sponsorship.

## INTRODUCTION

In Latin America and the Caribbean, one in four adults are obese and chronic non-communicable diseases are responsible for three out of every four deaths^1^. In parallel, sales of ultra-processed foods (UPF) have grown in all countries of this region^2^, and the average annual *per capita* consumption of alcohol in the continent is the second highest in the world^3^. This epidemiological scenario is favored by the marketing of UPFs and alcoholic beverages, promoted, among others, by the sponsorship of sports organizations^4^.

Sponsorship by UPF and alcoholic beverage companies in the sports scene has been described in Brazil^5^, Argentina^6^, the United States^7^, New Zealand^8^, Australia^9^, and in European countries^10^. These companies use sponsorship as a way to expose their brands, printed on uniforms, banners, and scoreboards on the field during a game, for product licensing and naming rights, and to promote advertisements during television broadcasts, etc.^4 , 11^

Sports sponsorship is a recurrent and attractive practice for sponsoring companies, which achieve greater recognition of their brands by the fan public and thus influence the consumers’ buying decision^12^. This practice is also interesting for federations and sports clubs, which increase their revenues and gain more visibility^8^. However, from a public health point of view, sports sponsorship by UPF and alcoholic beverage companies is seen as critical, since it is a violation of the human right to adequate food and promotes health externalities^4^.

It is important to pay special attention to the abusive character of this practice when directed to children and adolescents, due to the greater vulnerability of this public to recognize the commercial appeal of sponsorship. Thus, the effects of exposure of children and adolescents to sports marketing by UPF and alcoholic beverage companies is more pronounced, putting them at greater risk of consuming these products^13 , 14^. As a way to reduce this commercial practice and aiming to ensure the fulfillment of the human right to adequate food, the regulation of sports marketing is pointed out as a dimension to be worked on^15^.

In Latin America and the Caribbean, especially for countries in the Southern region where sports competitions are frequent, understanding how commercial partnerships between sponsoring companies and sporting organizations take place is an important step in designing actions to restrict food and beverage marketing and to reduce consumption of UPFs and alcoholic beverages on the continent.

This study aims to advance this field of knowledge by identifying the profile of companies in the food and beverage industry that sponsor the teams participating in the 2019 Copa América, the world’s oldest national soccer tournament and the largest competition among national teams in South America^16^, as well as associating the occurrence of this sponsorship with characteristics of the teams and their respective countries.

## METHODS

Exploratory study of sponsors of the 10 South American teams participating in the 46th edition of the Copa America Soccer Cup in 2019, listed below in alphabetical order: Argentina, Bolivia, Brazil, Chile, Colombia, Ecuador, Paraguay, Peru, Uruguay, and Venezuela.

The official sponsors were identified on the official websites of the soccer teams in August 2019 under the terminologies of official sponsors, partners, supporters, collaborators or suppliers. These entities were further classified into (i) food companies (food brands and food establishment) (ii) alcoholic beverages and (iii) other segments (consumer goods, communication and finance).

The food company category was sub-classified according to the food profile most predominantly offered^5^. For this the NOVA^17^ classification was used, which considers nature, extent, purpose, and degree of industrial processing of food. Thus, the foods were identified as: fresh, minimally processed, and ultra-processed (UPF). UPFs were further sub-classified into: ultra-processed beverages and other UPFs. No brands of culinary ingredients or processed products were identified among the companies sponsoring the teams.

The sponsorship data were associated with the characteristics of the teams and their respective countries, including: (i) number of titles won in previous editions of Copa América and World Cup, according to information obtained from the official website of Copa América and in websites of journalistic soccer content (ii) Human Development Index (HDI)^18^; (iii) annual *per capita* sales of UPF (kg)^2^; and (iv) annual *per capita* consumption of alcoholic beverages (L of pure ethanol)^3^.

The data analysis included the description, in absolute and relative frequency, of the sponsoring companies, according to product subcategories, and of the variables that characterize the teams/countries (stratified in medians). Pearson’s correlation test was applied to assess correlation between the characteristics of the teams and the percentage of sponsorship by food and UPF-specific companies at the 5% significance level (p < 0.05). The data were tabulated in Excel and analyzed in Stata software version 12.0.

## RESULTS

A total of 89 sponsorships were identified for the 10 teams studied, some of them sharing the same sponsor; 18.0% (n = 16) were from food companies, with 3.4% (n = 3) from fresh or minimally processed foods and 2.2% (n = 2) from food establishments. UPF firms were most prevalent in the food segment (n = 11; 12.4%), with the ultra-processed beverage category (n = 10; 11.2%) standing out. The alcoholic beverage category accounted for 7.9% (n = 7) of the sponsorships, and other sponsorship segments were noted in 74.2% (n = 66) of the sample.

The teams from Uruguay, Peru, and Argentina had the highest participation of sponsoring food companies: 33.3% (n = 3), 28.6% (n = 2), and 26.3% (n = 5), respectively. Ecuador stood out as the team with the highest percentage of sponsorship by alcoholic beverage companies (n = 1; 25.0%) ( Table 1 ).

**Table 1:** Sponsorship characteristics, title history, ultra-processed food market, alcohol consumption, and socioeconomic development of the teams’ countries. Copa América, 2019.

National teams	Sponsorship – n (%)	Sponsoring company - n (%)			National team titles - n		General characteristics of the countries		
		Food^a^	Alcoholic beverages	Other	Copa América 2019	World Cup	HDI	UPF sale^b^	Consumption of alcoholic beverages^c^
Argentina	19 (21.4)	5 (26.3)	1 (5.3)	13 (68.4)	14	2	0.83	185.0	9.8
Brazil	14 (15.7)	2 (14.3)	0 (0.0)	12 (85.7)	9	5	0.76	112.9	7.8
Paraguay	13 (14.6)	2 (15.4)	1 (7.7)	10 (76.9)	2	0	0.72	-	7.2
Colombia	11 (12.4)	1 (9.1)	1 (9.1)	9 (81.8)	1	0	0.76	90.2	5.8
Uruguay	9 (10.1)	3 (33.3)	1 (11.1)	5 (55.6)	15	2	0.81	149.5	10.8
Bolivia	7 (7.9)	1 (14.3)	1 (14.3)	5 (71.4)	1	0	0.70	102.5	5.5
Peru	7 (7.9)	2 (28.6)	1 (14.3)	4 (57.1)	2	0	0.76	83.2	6.3
Chile	4 (4.5)	0 (0.0)	0 (0.0)	4 (100.0)	2	0	0.85	201.9	9.3
Ecuador	4 (4.49)	0 (0.0)	1 (25.0)	3 (75.0)	0	0	0.76	88.0	4.4
Venezuela	1 (1.1)	0 (0.0)	0 (0.0)	1 (100.0)	0	0	0.76	-	5.6
**Total**	**89**	**16**	**7**	**66**	-	-	-	-	-

HDI: Human Development Index; UPF: ultra-processed foods.

^a ^We chose not to break down the category of sponsorship by food companies in order to obtain a general characterization of the sponsor profile of this segment by selection, besides the low participation of the categories of fresh and minimally processed foods and food establishment.

^b ^Annual *per capita* sales of ultra-processed foods in kilograms.

^c ^Annual *per capita* consumption of alcoholic beverages in liters of pure ethanol.

Ultra-processed beverage and alcoholic beverage companies sponsored seven of the 10 teams studied. For this first category, Coca-Cola was the company that sponsored the most teams: five out of the 10 studied. As for alcoholic beverages, the beer segment was the most prevalent, sponsoring six of the 10 teams studied ( Table 2 ).

**Table 2:** Description of the food and alcoholic beverage companies sponsoring the teams. Copa América, 2019.

Sponsors	Company segment	Sponsored national teams	
		Identification	n
Unprocessed or minimally processed foods			
Bonaqua	Mineral water	Argentina	1
Pechugon	Frozen poultry	Paraguay	1
Lavaggi	Pasta	Peru	1
Ultra-processed foods			
Ultra-processed beverages			
Coca-Cola	Soda	Argentina, Bolivia, Paraguay, Peru, Uruguay	5
Powerade	Isotonic drink	Argentina, Uruguay	2
Três Corações	Powder for preparing caffeinated drinks	Brazil	1
Guaraná Antárctica	Soda	Brazil	1
Colombiana	Soda	Colombia	1
Other ultra-processed			
Fargo	Bakery products	Argentina	1
Food establishment			
Mostaza	*Fast-food*	Argentina	1
Ta-Ta Supermercados	Supermarket	Uruguay	1
Alcoholic beverages			
Pilsen	Beer	Paraguay, Uruguay	2
Toro	Wine	Argentina	1
Paceña	Beer	Bolivia	1
Aguila	Beer	Colombia	1
Cerveza Pilsener	Beer	Ecuador	1
Cristal	Beer	Peru	1

We noticed a higher participation of sponsoring food companies, especially UPF, in teams from countries with higher HDI, UPF sales, and number of World Cup ( Table 3 ) and Copa America ( Table 3 and Figure ) titles. The sponsorship by alcoholic beverage companies was higher for teams from countries with lower HDI, alcohol consumption, and number of Copa América and World Cup titles ( Table 4 ).

**Table 3:** Characteristics of teams and countries, according to sponsorship by food companies. Copa América, 2019.

Features	Sponsorship by food companies (%)	Sponsorship by type of food company (%)	
		Fresh or MP	UPF
HDI			
Median 1 (0.70–0.75)	11.7	4.4	7.3
Median 2 (0.76–0.85)	16.6	1.1	12.3
Annual per capita sales of UPF (Kg)			
Median 1 (83.2–102.5)	10.4	2.9	7.5
Median 2 (112.9–201.9)	18.5	1.3	13.1
Copa América Titles			
Median 1 (0–2)	9.6	3.1	6.5
Median 2 (9–15)	24.6	1.8	17.4
World Cup Titles			
Median 1 (0)	9.6	3.1	6.5
Median 2 (2–5)	24.6	1.8	17.4

HDI: Human Development Index; UPF: ultra-processed foods. MP: minimally processed foods.

**Figure:**
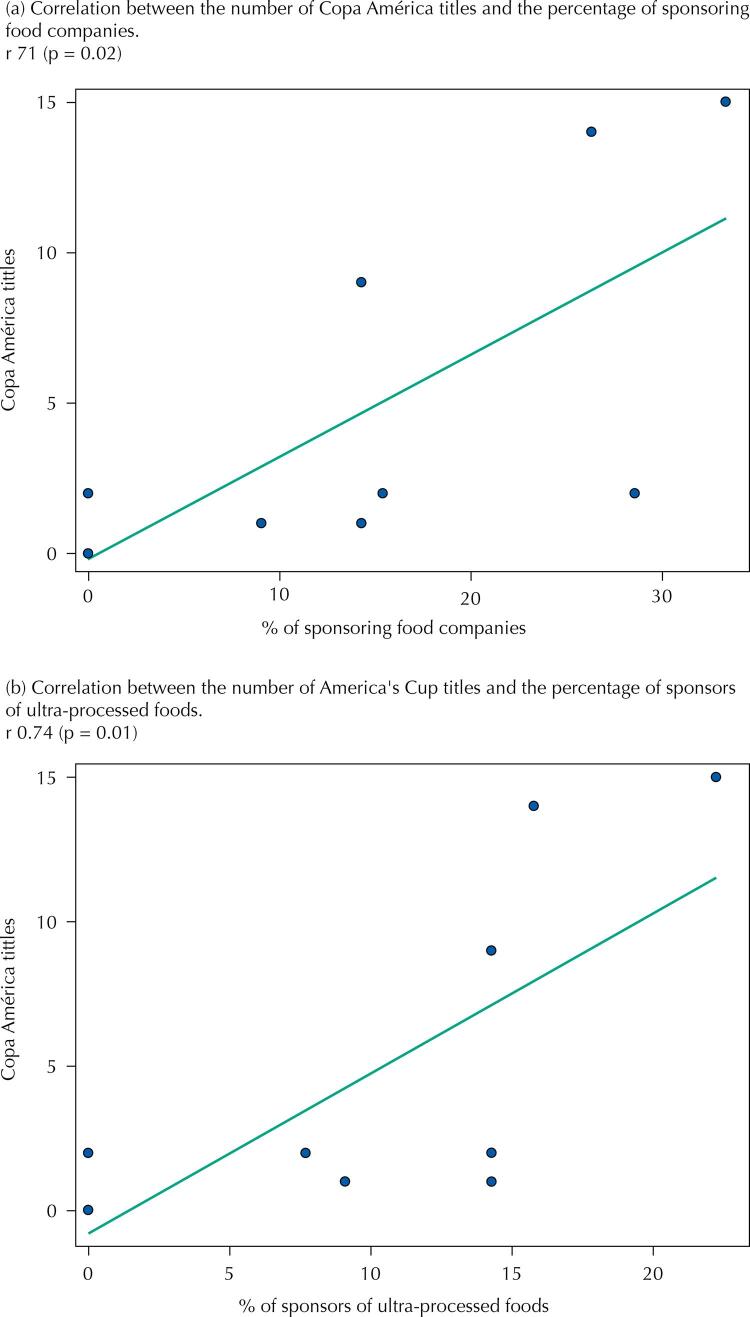
Relationship between the percentage of food company sponsorships and Copa América titles, 2019.

**Table 4:** Characteristics of teams and countries, according to sponsorship by alcoholic beverage companies. Copa América, 2019.

Features	Sponsorship by alcoholic beverage companies (%)
HDI	
Median 1 (0.70–0.75)	12.3
Median 2 (0.76–0.85)	5.1
Annual per capita consumption of alcoholic beverages (L of pure ethanol)	
Median 1 (4,4–6,3)	12.5
Median 2 (7,2–10,8)	4.8
Copa América Titles	
Median 1 (0–2)	10.1
Median 2 (9–15)	5.5
World Cup Titles	
Median 1 (0)	10.1
Median 2 (2–5)	5.5

HDI: Human Development Index.

## DISCUSSION

This study reveals the high participation of food and alcoholic beverage companies in sports sponsorships of the teams participating in the 2019 Copa América Soccer Cup and signals characteristics associated with this practice. In this sense, we noticed a higher participation of sponsorship by UPF companies among teams with better sports performance history and from countries with higher socioeconomic development and higher participation in the ultra-processed food market. On the other hand, support from alcoholic beverage companies was higher among teams with a lower sports performance record and from countries with lower socioeconomic development and alcoholic beverage market share.

The high presence of UPF companies as sports sponsors has been documented in studies conducted in various parts of the world^5^. Among Latin American countries, in Brazil, food companies represent 13.5% of the total sponsorships of soccer clubs, with 9.4% being UPF companies^5^. In Argentina, the country’s leading soft drink company sponsors children’s sporting events and distributes its products to the public during the tournaments^6^. In countries outside of Latin America, a recent systematic review showed that both children and adults are frequently exposed to marketing of unhealthy foods and beverages through sports sponsorship^4^.

Among the ultra-processed beverage brands that sponsored the teams participating in Copa América 2019, Coca-Cola was the leader in the number of teams sponsored. This brand has a tradition in sports sponsorship, as it has also supported the Olympics since 1928^7^and the International Federation of Football Associations (FIFA) World Cup since 1978^19^. In these competitions, the brand invests large amounts of money in the sports clubs and in different marketing actions^7^.

This study also reveals that the commercial partnership between sponsors and teams is not random, and teams from countries with higher UPF sales volume and better socioeconomic development are more often sponsored by ultra-processed food companies. Local market demand for UPF can justify these relationships. Argentina and Uruguay, for example, are countries with better socioeconomic development, with higher UPF^2^ sales *per capita* , and their national teams have the highest percentage of food sponsorships in South America. In this context, the strategic involvement of UPF companies in sports sponsorship is pointed out, in order to maintain their space in the local market, taking advantage of the context of favoritism and devotion to the sport to promote themselves.

Furthermore, it was found that the decision of UPF brands to sponsor teams also involves the history of winning competitions, which is directly related to the popularity of the team^5^. The national teams of Argentina, Uruguay, and Brazil have the longest history of winning championships in South America and are among the top 10 teams in the world in competition performance^20^. Thus, the sponsorship practiced by UPF companies in this context is also strategic, due to the broad brand visibility provided by these sports organizations compared to those of lower performance.

The study also shows the large participation of alcoholic beverage companies as sponsors of South American teams, especially for the beer segment. In contrast to sponsorship by UPF companies, alcoholic beverage companies are focused on teams from countries with lower socioeconomic development and lower *per capita* alcohol consumption. In fact, in the last decades, the alcohol industry has expanded in many low- and middle-income countries, mainly through its influence on local governments to minimize impacts and prevent the creation of policies to control and prevent its abusive consumption. Moreover, in these countries, large alcoholic beverage brands develop corporate social responsibility practices with the intention of camouflaging their externalities on the populations’ health^21^.

Thus, given the influence of sports sponsorship on the consumption of health-damaging products, regulation of this practice is necessary to reduce its impact on public health^4^. In terms of marketing of unhealthy foods, some of these countries, such as Peru, Brazil and Chile, have legislation that limits its targeting to children or that makes it mandatory to link a warning to the advertising piece about the risk of excessive consumption of unhealthy foods in different media. However, in none of the regulatory codes of these countries are there any restrictions on the association of unhealthy food companies with sports sponsorship^22^.

Regarding the marketing of alcoholic beverages, Argentina and Chile have laws that impose restrictions on the content of advertising messages directed at minors that encourage abusive consumption. However, there is also no restriction on the practice of sports sponsorship by these companies^23 , 24^. Brazil, on the other hand, has a more restrictive legislation compared to the previous countries, since it prohibits, in the advertising of these drinks, their association with Olympic or competition sports. Nevertheless, as far as sports sponsorship is concerned, there is a ban only on advertising beverages with an alcohol content above 13 degrees *Gay Lussac* ; that is, beverages with lower alcohol content, such as beer and wine, are not regulated^25^.

In this sense, it is necessary to increase the scope of these laws to limit the sponsorship of cultural and sporting events by unhealthy food and beverage companies, and to promote the replacement of these sponsors by companies with healthier product portfolios, minimizing the negative impacts of the former on the consumer public^11^. In parallel, the advancement of this agenda also involves limiting the benefits that companies achieve by executing sponsorship actions, discouraging this practice. In Brazil, for example, tax benefits were granted to sponsors of the 2016 Olympic Games^26 , 27^.

However, restrictions on the marketing of ultra-processed foods and alcoholic beverages face strong opposition from companies, which *lobby* in Congress and regulatory agencies to block the implementation of regulatory policies. They use as argument information about the economic importance of these sectors for the generation of jobs and for the Gross Domestic Product (GDP) of countries, without also presenting the costs of externalities and health impacts associated with the consumption of these products by the population^28^. In this sense, it is worth exemplifying the tobacco control agenda in Brazil, which, despite having presented advances, faced significant interference from the industry in regulatory actions, using the fallacy of the impact of national tobacco production as an opportunity for Brazil in foreign trade to try to influence political systems^29^.

Despite the novelty and the potential implications of the study results, some limitations are highlighted, such as: the exclusive analysis of the presence of the sponsors’ logos on the teams’ official websites, without evaluating other strategies, such as the sponsored contents of the teams’ social media, for example. In addition, it was not possible to obtain other data about the teams, such as the amount received by each of the identified sponsors and the number of consumers reached by the sponsorship, characteristics that may also influence the practice of sponsorship. Nevertheless, the results obtained describe, for the first time, the sponsorship profile of food and beverage companies in South American soccer and the sport performance, socioeconomic, and market characteristics that potentially influence the companies’ sponsorship decision.
