# Supplementary material for: Sponsorship by food and beverage companies in soccer: an analysis of the 2019 Copa América
Source: Rev Saude Publica. 2022 May 18;56:41. doi: 10.11606/s1518-8787.2022056003491 (PMC9126579; doi:10.11606/s1518-8787.2022056003491)
Supplement: Translation

# Translation: Patrocínio por empresas de alimentos e bebidas no futebol: análise da Copa América 2019

**Authors:** Larissa Cardoso de Miranda Araujo, Juliana de Paula Matos, Paula Martins Horta

**Affiliations:** I Brasil Universidade Federal de Minas Gerais. Departamento de Nutrição. Belo Horizonte, MG, Brasil

**Keywords:** Futebol, Apoio Financeiro, Indústria Alimentícia, Alimentos e Bebidas, Marketing

## Abstract

**OBJETIVO:**

Identificar o patrocínio por empresas do setor de alimentos e bebidas das seleções participantes da Copa América de Futebol 2019 e associar esse patrocínio a características das seleções e de seus respectivos países.

**MÉTODOS:**

Foram identificados os patrocinadores das 10 seleções participantes da 46ª edição da Copa América de Futebol. Essas entidades foram classificadas em empresas i) de alimentos, ii) de bebidas alcoólicas e iii) de outros segmentos. As empresas de alimentos foram classificadas de acordo com seus produtos, segundo a classificação NOVA. Além disso, dados sobre o número de títulos anteriormente conquistados pelas seleções na Copa América e na Copa do Mundo foram obtidos, assim como dados do índice de desenvolvimento humano, de venda anual *per capita* de alimentos ultraprocessados e de consumo anual *per capita* de bebidas alcoólicas dos países.

**RESULTADOS:**

Um total de 89 patrocínios foram identificados para as 10 seleções estudadas, alguns desses apoiando duas ou mais equipes. Dezoito por cento dos patrocinadores eram empresas de alimentos, sendo 12,4% de alimentos ultraprocessados. A categoria de bebidas alcoólicas representou 7,9% dos patrocinadores. As empresas de bebidas ultraprocessadas e as de bebidas alcoólicas patrocinaram sete das 10 seleções estudadas. Notou-se maior participação de patrocinadores de empresas de alimentos ultraprocessados em seleções de países com maior índice de desenvolvimento humano, venda de alimentos ultraprocessados e número de títulos de Copa América e Copa do Mundo. Já o patrocínio por empresas de bebidas alcoólicas foi maior em seleções de países com menor índice de desenvolvimento humano, consumo de álcool e número de títulos em Copa América e Copa do Mundo.

**CONCLUSÃO:**

Evidenciou-se uma presença significativa de empresas de alimentos ultraprocessados e de bebidas alcoólicas como patrocinadores das seleções sul-americanas de futebol e que as características de desempenho esportivo das seleções e questões socioeconômicas e de mercado dos países se associam à ocorrência do patrocínio.

## INTRODUÇÃO

Na América Latina e Caribe, um em cada quatro adultos estão obesos e as doenças crônicas não transmissíveis são responsáveis por três de cada quatro mortes^1^. Em paralelo, as vendas de alimentos ultraprocessados (AUP) cresceram em todos os países dessa região^2^e o consumo *per capita* anual médio de álcool no continente é o segundo maior no mundo^3^. Esse cenário epidemiológico é favorecido pelo marketing de AUP e bebidas alcoólicas, promovido, entre outros, pelo patrocínio a organizações esportivas^4^.

O patrocínio por empresas de AUP e bebidas alcoólicas no cenário esportivo já foi descrito no Brasil^5^, Argentina^6^, Estados Unidos^7^, Nova Zelândia^8^, Austrália^9^e em países europeus^10^. Essas empresas utilizam o patrocínio como forma de expor suas marcas, estampadas em uniformes, banners e placares no campo durante um jogo, para licenciamento de produtos e direitos de nome e para promover anúncios durante transmissão televisiva etc.^4 , 11^

O patrocínio esportivo é uma prática recorrente e atrativa para as empresas patrocinadoras, que alcançam maior reconhecimento de suas marcas pelo público torcedor e, com isso, influenciam a decisão de compra dos consumidores^12^. Essa prática também é interessante para as organizações federativas e clubes esportivos, que aumentam sua receita e ganham maior visibilidade^8^. Porém, do ponto de vista de saúde pública, o patrocínio esportivo por empresas de AUP e bebidas alcoólicas é tido como crítico, uma vez que constitui uma violação ao direito humano à alimentação adequada e promove externalidades em saúde^4^.

É importante dar atenção especial ao caráter abusivo dessa prática, quando direcionada a crianças e adolescentes, devido à maior vulnerabilidade desse público em reconhecer o apelo comercial do patrocínio. Com isso, os efeitos da exposição de crianças e adolescentes ao marketing esportivo de empresas de AUP e bebidas alcoólicas é mais pronunciado, colocando-os sob maior risco do consumo desses produtos^13 , 14^. Como forma de reduzir essa prática comercial e visando garantir o cumprimento do direito humano à alimentação adequada , aponta-se a regulamentação do marketing esportivo como uma dimensão a ser trabalhada^15^.

Na América Latina e Caribe, principalmente para os países da região sul, onde competições esportivas são frequentes, compreender como ocorrem as parcerias comerciais entre as empresas patrocinadoras e as organizações esportivas é um passo importante para o delineamento de ações de restrição ao marketing de alimentos e bebidas e que almejam reduzir o consumo de AUP e de bebidas alcoólicas no continente.

O presente estudo objetiva avançar nesse campo de conhecimento ao identificar o perfil de empresas do setor de alimentos e bebidas que patrocinam as seleções participantes da Copa América de Futebol 2019, o torneio de seleções mais antigo do mundo e a maior competição entre seleções da América do Sul^16^, além de associar a ocorrência desse patrocínio a características das seleções e de seus respectivos países.

## MÉTODOS

Estudo exploratório dos patrocinadores das 10 seleções sul-americanas participantes da 46ª edição da Copa América de Futebol em 2019, listadas a seguir em ordem alfabética: Argentina, Bolívia, Brasil, Chile, Colômbia, Equador, Paraguai, Peru, Uruguai e Venezuela.

Os patrocinadores oficiais foram identificados nos sites oficiais das seleções de futebol em agosto de 2019, sob as terminologias de patrocinadores, parceiros, apoiadores, colaboradores ou fornecedores oficiais. Essas entidades foram posteriormente classificadas em empresas i) de alimentos (marcas de alimentos e estabelecimento de alimentos) ii) de bebidas alcoólicas e iii) de outros segmentos (bens de consumo, comunicação e finanças).

A categoria empresas de alimentos foi subclassificada, de acordo com o perfil de alimento mais predominantemente ofertado^5^. Para isso foi utilizada a classificação NOVA^17^, que considera natureza, extensão, propósito e grau de processamento industrial dos alimentos. Dessa forma, os alimentos foram identificados em: *in natura* e minimamente processados e AUP. Os AUP foram ainda subclassificados em: bebidas ultraprocessadas e outros AUP. Não foram identificadas marcas de ingredientes culinários ou produtos processados entre as empresas patrocinadoras das seleções.

Os dados de patrocínio foram associados às características das seleções e de seus respectivos países, incluindo: i) número de títulos conquistados em edições anteriores da Copa América e da Copa do Mundo, segundo informações obtidas do site oficial da Copa América e em sites de conteúdo jornalístico de futebol; ii) Índice de Desenvolvimento Humano (IDH)^18^; iii) venda anual *per capita* de AUP (kg)^2^e iv) consumo anual *per capita* de bebidas alcoólicas (L de etanol puro)^3^.

A análise dos dados contemplou a descrição, em frequência absoluta e relativa, das empresas patrocinadoras, segundo as subcategorias de produtos, e das variáveis de caracterização das seleções/países (estratificadas em medianas). Aplicou-se teste de correlação de Pearson para relacionar as características das seleções e o percentual de patrocínio das empresas de alimentos e de modo específico de AUP ao nível de significância de 5% (p < 0,05). Os dados foram tabulados em Excel e analisados no software Stata versão 12.0.

## RESULTADOS

Um total de 89 patrocínios foram identificados para as 10 seleções estudadas, algumas delas dividindo o mesmo patrocinador; 18,0% (n = 16) eram de empresas de alimentos, sendo 3,4% (n = 3) de alimentos *in natura* ou minimamente processados e 2,2% (n = 2) de estabelecimento de alimentos. As empresas de AUP foram as mais prevalentes no segmento de alimentos (n = 11; 12,4%), com destaque para a categoria de bebidas ultraprocessadas (n = 10; 11,2%). A categoria de bebidas alcoólicas representou 7,9% (n = 7) dos patrocínios e outros segmentos de patrocínio foram notados em 74,2% (n = 66) da amostra.

As seleções do Uruguai, Peru e Argentina foram as que apresentaram maior participação de empresas de alimentos patrocinadoras: 33,3% (n = 3), 28,6% (n = 2) e 26,3% (n = 5), respectivamente. Já o Equador destacou-se por ser a seleção com o maior percentual de patrocínio por empresas de bebidas alcoólicas (n = 1; 25,0%) ( Tabela 1 ).

**Tabela 1:** Características de patrocínio, histórico de títulos, mercado de alimentos ultraprocessados, consumo de bebidas alcoólicas e desenvolvimento socioeconômico dos países das seleções. Copa América, 2019.

Seleções	Patrocínio – n (%)	Empresa patrocinadora – n (%)			Títulos das seleções – n		Características gerais dos países		
		Alimentos^a^	Bebidas alcoólicas	Outros	Copa América	Copa do Mundo	IDH	Venda de AUP^b^	Consumo de bebidas alcoólicas^c^
Argentina	19 (21,4)	5 (26,3)	1 (5,3)	13 (68,4)	14	2	0,83	185,0	9,8
Brasil	14 (15,7)	2 (14,3)	0 (0,0)	12 (85,7)	9	5	0,76	112,9	7,8
Paraguai	13 (14,6)	2 (15,4)	1 (7,7)	10 (76,9)	2	0	0,72	-	7,2
Colômbia	11 (12,4)	1 (9,1)	1 (9,1)	9 (81,8)	1	0	0,76	90,2	5,8
Uruguai	9 (10,1)	3 (33,3)	1 (11,1)	5 (55,6)	15	2	0,81	149,5	10,8
Bolívia	7 (7,9)	1 (14,3)	1 (14,3)	5 (71,4)	1	0	0,70	102,5	5,5
Peru	7 (7,9)	2 (28,6)	1 (14,3)	4 (57,1)	2	0	0,76	83,2	6,3
Chile	4 (4,5)	0 (0,0)	0 (0,0)	4 (100,0)	2	0	0,85	201,9	9,3
Equador	4 (4,49)	0 (0,0)	1 (25,0)	3 (75,0)	0	0	0,76	88,0	4,4
Venezuela	1 (1,1)	0 (0,0)	0 (0,0)	1 (100,0)	0	0	0,76	-	5,6
**Total**	**89**	**16**	**7**	**66**	-	-	-	-	-

IDH: índice de desenvolvimento humano; AUP: alimentos ultraprocessados.

^a^Optou-se por não desmembrar a categoria de patrocínio por empresas de alimentos com o objetivo de obter uma caracterização geral do perfil de patrocinador desse segmento por seleção, além da baixa participação das categorias de alimentos *in natura* e minimamente processados e estabelecimento de alimentos.

^b^Venda anual *per capita* de alimentos ultraprocessados em quilogramas.

^c^Consumo anual *per capita* de bebidas alcoólicas em litros de etanol puro.

As empresas de bebidas ultraprocessadas e as de bebidas alcoólicas patrocinaram sete das 10 seleções estudadas. Para essa primeira categoria, a Coca-Cola foi a empresa que mais patrocinou seleções: cinco das 10 estudadas. Já para as bebidas alcoólicas, o segmento de cervejas foi o mais prevalente, patrocinando seis das 10 seleções estudadas ( Tabela 2 ).

**Tabela 2:** Descrição das empresas de alimentos e bebidas alcoólicas patrocinadoras das seleções. Copa América, 2019.

Patrocinadores	Segmento de empresa	Seleções patrocinadas	
		Identificação	n
Alimentos *in natura* ou minimamente processados			
Bonaqua	Água mineral	Argentina	1
Pechugon	Aves congeladas	Paraguai	1
Lavaggi	Massas	Peru	1
Alimentos ultraprocessados			
Bebidas ultraprocessadas			
Coca-Cola	Refrigerante	Argentina, Bolívia, Paraguai, Peru, Uruguai	5
Powerade	Bebida isotônica	Argentina, Uruguai	2
Três Corações	Pó para preparo de bebidas cafeinadas	Brasil	1
Guaraná Antárctica	Refrigerante	Brasil	1
Colombiana	Refrigerante	Colômbia	1
Outros ultraprocessados			
Fargo	Produtos de panificação	Argentina	1
Estabelecimento de alimentos			
Mostaza	Rede de *fast-food*	Argentina	1
Ta-Ta Supermercados	Supermercado	Uruguai	1
Bebidas alcoólicas			
Pilsen	Cerveja	Paraguai, Uruguai	2
Toro	Vinho	Argentina	1
Paceña	Cerveja	Bolívia	1
Aguila	Cerveja	Colômbia	1
Cerveza Pilsener	Cerveja	Equador	1
Cristal	Cerveja	Peru	1

Notou-se maior participação de empresas de alimentos patrocinadoras, sobretudo de AUP, em seleções de países com maior IDH, venda de AUP e número de títulos de Copa do Mundo ( Tabela 3 ) e Copa América ( Tabela 3 e Figura ). Já o patrocínio por empresas de bebidas alcoólicas foi maior em seleções de países com menor IDH, consumo de álcool e número de títulos em Copa América e Copa do Mundo ( Tabela 4 ).

**Tabela 3:** Características das seleções e países, segundo patrocínio por empresas de alimentos. Copa América, 2019.

Características	Patrocínio por empresas de alimentos (%)	Patrocínio por tipo de empresa de alimentos (%)	
		In natura ou MP	AUP
IDH			
Mediana 1 (0,70–0,75)	11,7	4,4	7,3
Mediana 2 (0,76–0,85)	16,6	1,1	12,3
Venda anual per capita de AUP (Kg)			
Mediana 1 (83,2–102,5)	10,4	2,9	7,5
Mediana 2 (112,9–201,9)	18,5	1,3	13,1
Títulos em Copa América			
Mediana 1 (0–2)	9,6	3,1	6,5
Mediana 2 (9–15)	24,6	1,8	17,4
Títulos em Copa do Mundo			
Mediana 1 (0)	9,6	3,1	6,5
Mediana 2 (2–5)	24,6	1,8	17,4

IDH: índice de desenvolvimento humano; AUP: alimentos ultraprocessados; MP: minimamente processados.

**Figura:** Relação entre o percentual de patrocinadores de empresas de alimentos e títulos em Copa América, 2019.

**Tabela 4:** Características das seleções e países, segundo patrocínio por empresas de bebidas alcoólicas. Copa América, 2019.

Características	Patrocínio por empresas de bebidas alcoólicas (%)
IDH	
Mediana 1 (0,70–0,75)	12,3
Mediana 2 (0,76–0,85)	5,1
Consumo anual per capita de bebidas alcoólicas (L de etanol puro)	
Mediana 1 (4,4–6,3)	12,5
Mediana 2 (7,2–10,8)	4,8
Títulos em Copa América	
Mediana 1 (0–2)	10,1
Mediana 2 (9–15)	5,5
Títulos em Copa do mundo	
Mediana 1 (0)	10,1
Mediana 2 (2–5)	5,5

IDH: índice de desenvolvimento humano.

## DISCUSSÃO

Este estudo revela a elevada participação de empresas de alimentos e de bebidas alcoólicas em patrocínios esportivos das seleções participantes da Copa América de Futebol 2019 e sinaliza características associadas à essa prática. Nesse sentido, notou-se maior participação do patrocínio por empresas de AUP entre as seleções com melhor histórico de desempenho esportivo e de países com maior desenvolvimento socioeconômico e maior participação no mercado de ultraprocessados. Por outro lado, o apoio de empresas de bebidas alcoólicas foi maior entre as seleções com menor histórico de desempenho esportivo e de países que apresentam menor desenvolvimento socioeconômico e participação no mercado de bebidas alcoólicas.

A elevada presença de empresas de AUP como patrocinadores esportivos já foi documentada em estudos conduzidos em diversas partes do mundo^5^. Entre os países da América Latina, no Brasil, as empresas de alimentos representam 13,5% do total de patrocinadores de clubes de futebol, sendo 9,4% de empresas de AUP^5^. Na Argentina, a principal empresa de refrigerantes do país patrocina eventos esportivos infantojuvenis e distribui seus produtos para o público durante os torneios^6^. Em países fora da América Latina, uma recente revisão sistemática mostrou que tanto crianças como adultos são expostos frequentemente ao marketing de alimentos e bebidas não saudáveis mediante o patrocínio no esporte^4^.

Dentre as marcas de bebidas ultraprocessadas que patrocinaram as seleções participantes da Copa América 2019, a Coca-Cola foi a líder no número de seleções patrocinadas. Essa marca tem tradição no patrocínio esportivo, uma vez que apoia também as Olimpíadas desde 1928^7^e a Copa do Mundo da Federação Internacional de Futebol (FIFA) desde 1978^19^. Nessas competições, a marca investe elevados montantes financeiros nos clubes esportivos e em diferentes ações de marketing^7^.

O presente estudo revela ainda que a parceria comercial entre os patrocinadores e as seleções não é aleatória e seleções de países com maior volume de venda de AUP e melhor desenvolvimento socioeconômico são mais patrocinadas por empresas de ultraprocessados. A demanda do mercado local por AUP pode justificar essas relações. A Argentina e o Uruguai, por exemplo, são países com melhor desenvolvimento socioeconômico, com maior venda *per capita* de AUP^2^e suas seleções possuem maior percentual de patrocínios de alimentos na América do Sul. Nesse contexto, aponta-se o estratégico envolvimento das empresas de AUP no patrocínio no esporte, para manutenção do seu espaço no mercado local, se valendo do contexto de favoritismo e devoção ao esporte para se promoverem.

Ademais, verificou-se que a decisão das marcas de AUP em patrocinar as seleções também envolve o histórico de vitórias em competições, fato que está diretamente relacionado à popularidade da seleção^5^. As seleções da Argentina, Uruguai e Brasil são as que possuem um maior histórico de vitórias em campeonatos na América do Sul e estão entre as 10 melhores do mundo em desempenho em competições^20^. Desse modo, o patrocínio praticado pelas empresas de AUP nesse contexto também é estratégico, pela ampla visibilidade da marca proporcionada por essas organizações esportivas se comparado às de desempenho inferior.

O estudo mostra ainda a grande participação de empresas do setor de bebidas alcoólicas como patrocinadoras das seleções sul-americanas, principalmente para o segmento de cervejas. De modo contrário ao patrocínio por parte de empresas de AUP, empresas do ramo de bebidas alcoólicas estão concentradas em seleções de países com menor desenvolvimento socioeconômico e com menor consumo *per capita* de álcool. De fato, nas últimas décadas, a indústria do álcool se expandiu em muitos países de baixa e média renda, sobretudo por sua influência sobre governos locais para minimizar impactos e impedir a criação de políticas de controle e prevenção ao seu consumo abusivo. Além disso, nesses países, grandes marcas de bebidas alcoólicas desenvolvem práticas de responsabilidade social corporativa na intenção de camuflar suas externalidades na saúde das populações^21^.

Dessa forma, dada a influência do patrocínio esportivo no consumo de produtos danosos à saúde, faz-se necessária a regulação dessa prática para diminuição dos seus impactos em saúde pública^4^. Em termos de marketing de alimentos não saudáveis, alguns desses países, como Peru, Brasil e Chile, possuem legislações que limitam o seu direcionamento à criança ou que obriga a vinculação de um alerta à peça publicitária quanto ao risco do consumo excessivo de alimentos não saudáveis em diferentes meios de comunicação. No entanto, em nenhum dos códigos de regulação desses países há restrições quanto a associação das empresas de alimentos não saudáveis ao patrocínio esportivo^22^.

No que diz respeito ao marketing de bebidas alcoólicas, a Argentina e o Chile possuem legislações que impõem restrições ao conteúdo das mensagens publicitárias direcionadas a menores e que incentivem o consumo abusivo. Porém, também não há restrição à prática de patrocínio esportivo por essas empresas^23 , 24^. Já o Brasil possui uma legislação mais restritiva comparada às dos países anteriores, uma vez que proíbe na publicidade dessas bebidas, a sua associação ao esporte olímpico ou de competição. Apesar disso, no que concerne ao patrocínio esportivo, há proibição somente sobre a publicidade de bebidas com teor alcoólico acima de 13 graus *Gay Lussac* ; ou seja, bebidas com menor teor alcoólico, como a cerveja e o vinho, não são reguladas^25^.

Nesse sentido, faz-se necessário aumentar o escopo dessas legislações para limitar o patrocínio por parte de empresas de alimentos e bebidas não saudáveis a eventos culturais e esportivos, além de promover a substituição desses patrocinadores por empresas com portfólio de produtos mais saudáveis, minimizando os impactos negativos dos primeiros frente ao público consumidor^11^. Em paralelo, o avanço nessa agenda também passa por limitar benefícios que as empresas alcançam ao executar ações de patrocínio, desestimulando essa prática. No Brasil, por exemplo, foram concedidos benefícios fiscais aos patrocinadores dos Jogos Olímpicos de 2016^26 , 27^.

No entanto, as restrições ao marketing de alimentos ultraprocessados e bebidas alcoólicas enfrentam forte oposição das empresas, que desempenham ações de *lobby* no Congresso e agências reguladoras para barrar a implementação de políticas regulatórias, utilizando como argumento informações sobre a importância econômica desses setores para a geração de empregos e para o Produto Interno Bruto (PIB) dos países, sem apresentar também os custos das externalidades e impactos em saúde associados ao consumo desses produtos pela população^28^. Nesse sentido, cabe exemplificar a agenda do controle do tabaco no Brasil, que apesar de ter apresentado avanços, enfrentou significativa interferência da indústria em ações regulatórias, usando da falácia do impacto da produção nacional do tabaco como oportunidade para o Brasil no comércio exterior para tentar influenciar os sistemas políticos^29^.

Apesar do ineditismo e das potenciais implicações dos resultados do estudo, algumas limitações são destacadas, como: a análise exclusiva da presença dos logotipos dos patrocinadores nos sites oficiais das seleções, sem avaliação de outras estratégias, como os conteúdos patrocinados das mídias sociais das seleções, por exemplo. Além disso, não foi possível obter outros dados sobre as seleções, como a quantia recebida por cada um dos patrocinadores identificados e o número de consumidores alcançados pelo patrocínio, características que também podem influenciar na prática do patrocínio. Apesar disso, os resultados obtidos descrevem, pela primeira vez, o perfil de patrocínio de empresas de alimentos e bebidas no futebol sul-americano e as características de desempenho esportivo, socioeconômicas e de mercado que potencialmente influenciam na decisão das empresas pelo patrocínio.
